# Admission-based predictors of in-hospital AKI in HFrEF: a single-centre retrospective study

**DOI:** 10.3389/fphys.2026.1928628

**Published:** 2026-10-12

**Authors:** Feng Dai, Xiaomin Li, Zihe Wang, Siyu Tan, Fengyang Han, Xiang Song

**Affiliations:** Department of Cardiology, The Fourth Affiliated Hospital of Harbin Medical University, Harbin, China

**Keywords:** acute kidney injury, cardiorenal axis, cardio-renal syndrome, heart failure, predictive model

## Abstract

**Objective:**

This study aimed to construct and internally validate a concise prediction model for in-hospital acute kidney injury (AKI) among patients admitted with heart failure with reduced ejection fraction (HFrEF). The model was limited to information available at hospital presentation.

**Methods:**

We retrospectively analyzed 1,071 HFrEF hospitalizations at the Fourth Affiliated Hospital of Harbin Medical University from September 2020 to September 2022. The outcome was incident AKI during the index admission, defined using a modified KDIGO (48-h serum creatinine) criterion, based on an increase of ≥26.5 µmol/L within 48 h. Candidate variables measured at admission were screened with least absolute shrinkage and selection operator (LASSO) logistic regression using the 1−SE rule. Bootstrap stability and clinical parsimony were then considered to preserve temporal validity and bedside usability. Internal validation was performed using 5-fold stratified out-of-fold cross-validation.

**Results:**

In-hospital AKI occurred in 118 of the 1,071 patients (11.0%). Five key on-admission clinical parameters were identified as the strongest predictors: age, furosemide-equivalent dose, hypotension on presentation, RDW, eGFR. In internal validation, the combination of these five strictly admission-based parameters achieved an AUC-ROC of 0.81 [95% bootstrap confidence interval (CI): 0.77–0.85], a precision-recall AUC (PR-AUC) of 0.47, a Brier score of 0.08, a calibration intercept of −0.02, and a calibration slope of 0.98. Decision curve analysis demonstrated clinical utility with a positive net benefit across most clinically plausible decision thresholds.

**Conclusion:**

A five-predictor model based entirely on admission data can provide interpretable early risk stratification for in-hospital AKI in patients hospitalized with HFrEF. By excluding post-admission treatment responses and weight change, the model reduces reverse-causality bias and may support renal safety decisions at the time of presentation.

## Introduction

Heart failure (HF) is an increasing global health burden and remains a major cause of morbidity, repeated hospitalization, and poor outcomes in older adults (Roger, 2021; Khan et al., 2024). During acute decompensated heart failure (ADHF), renal dysfunction often evolves alongside cardiac deterioration and becomes a key determinant of prognosis (Ronco and Di Lullo, 2017; McDonagh et al., 2021; Heidenreich et al., 2022). Patients with heart failure with reduced ejection fraction (HFrEF) are particularly vulnerable (Ronco et al., 2010; Ronco and Di Lullo, 2017). In this phenotype, venous congestion and impaired forward perfusion may both be prominent, creating conditions for cardiorenal syndrome type 1, in which acute cardiac decompensation is followed by acute kidney injury (AKI). Clinically, up to one-third of ADHF patients with reduced ejection fraction experience incident AKI, which is consistently linked to prolonged length of stay and a significantly higher hazard of death (Damman et al., 2007; Metra et al., 2008; Hoste et al., 2018).

The mechanisms underlying cardiorenal syndrome type 1 are not limited to low cardiac output. Although reduced cardiac output can lower glomerular filtration pressure, recent clinical evidence has emphasized systemic and renal venous congestion as major determinants of glomerular filtration rate (Damman et al., 2009; Mullens et al., 2009). Backward transmission of elevated central venous pressure increases renal interstitial pressure, compresses the renal microcirculation, and reduces the transglomerular hydrostatic gradient (Mullens et al., 2009; Ambrosy et al., 2013). Loop-diuretic decongestion may further complicate this process (Mullens et al., 2019).

In ADHF, serum creatinine changes are therefore difficult to interpret during decompensated HF. A transient creatinine rise during effective decongestion may reflect hemoconcentration, whereas persistent creatinine elevation associated with ongoing congestion may indicate true renal injury (Mullens et al., 2022). Standardized heart failure risk stratification scores, such as the ADHERE CART or GWTG-HF models, were primarily derived to predict all-cause mortality; recent evidence demonstrates that despite their mortality prediction utility, such scores perform poorly when applied to acute cardiorenal outcomes like AKI (Aykut et al., 2026). Simple bedside prediction tools could help clinicians identify patients who require closer renal monitoring before overt AKI develops (Collins et al., 2015; Moons et al., 2015). We therefore developed and internally validated a parsimonious admission-based model using only baseline variables available at presentation.

## Materials and methods

### Study design and population

This single-center retrospective cohort study included patients hospitalized for HFrEF at the Fourth Affiliated Hospital of Harbin Medical University. After eligibility review and data cleaning, 1,071 patients were included in the analytic dataset according to **Figure 1**. The cohort was derived from electronic medical records and included demographic characteristics, vital signs, cardiac status, laboratory indices, comorbidities, medications, and early in-hospital treatment-response variables. The study followed the logic of transparent reporting of a multivariable prediction model and was interpreted with attention to Prediction Model Risk of Bias Assessment Tool (PROBAST) domains (Collins et al., 2015; Moons et al., 2015; Moons et al., 2019).

**Figure 1:**
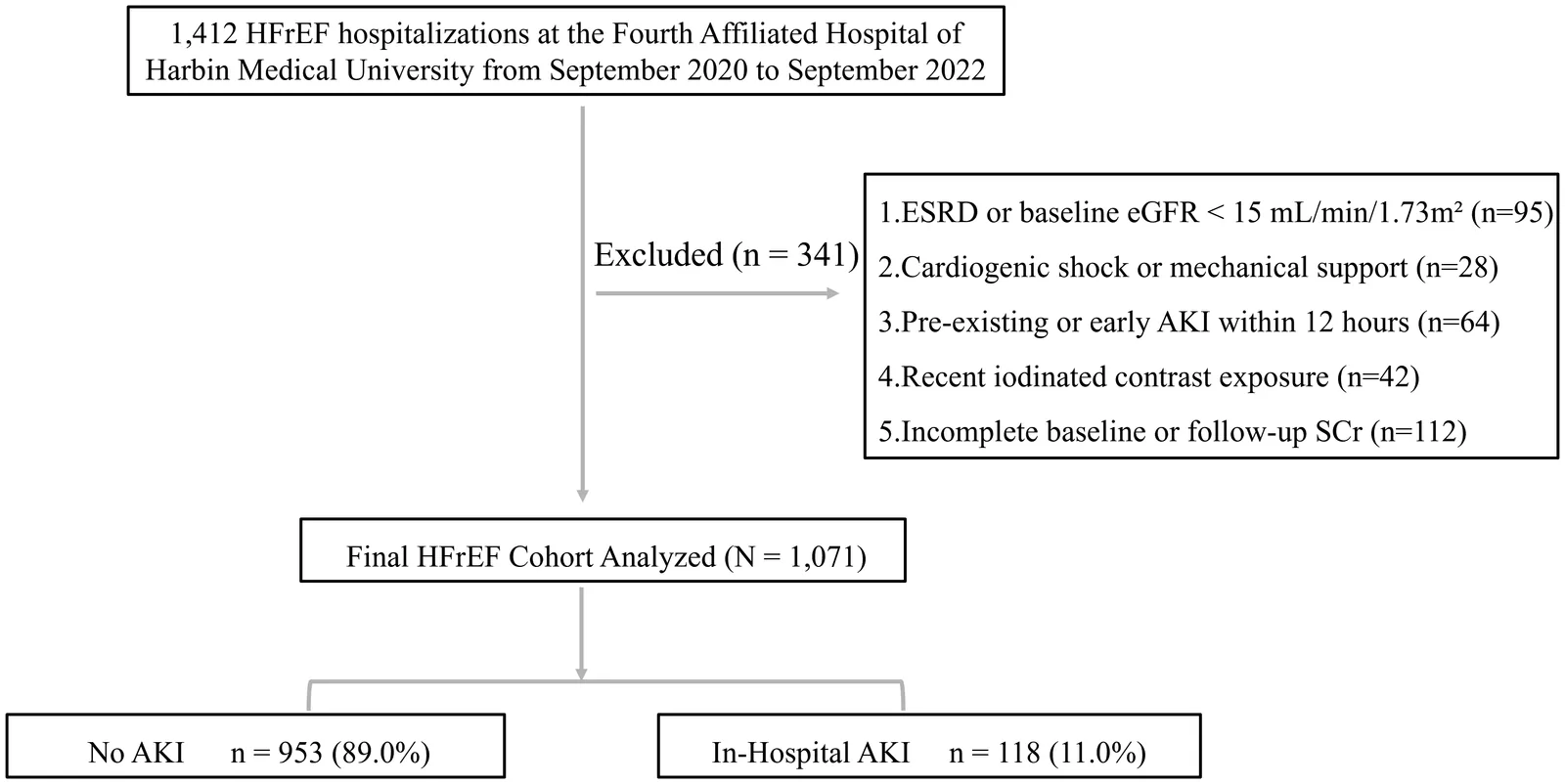
Study flow. HFrEF, heart failure with reduced ejection fraction; AKI, acute kidney injury; ESRD, end-stage renal disease.

The inclusion criterion was hospitalization with HFrEF during the source study period. HFrEF was diagnosed by attending cardiologists according to the contemporary guidelines of the European Society of Cardiology (ESC) (McDonagh et al., 2021) and the American College of Cardiology/American Heart Association (ACC/AHA) (Heidenreich et al., 2022). The diagnosis required typical symptoms and/or signs of HF (NYHA class II–IV), objective evidence of reduced LVEF (<40%), and elevated natriuretic peptides at admission. Initially, a total of 1,412 patients hospitalized with heart failure were screened. Of these, 341 patients were excluded based on the following pre-specified criteria: (1) severe chronic renal impairment, defined as end-stage renal disease (ESRD) or baseline estimated glomerular filtration rate (eGFR) < 15 mL/min/1.73 m² (*n* = 95); (2) cardiogenic shock or emergency mechanical circulatory support on admission (*n* = 28); (3) pre-existing or early-onset acute kidney injury (AKI) diagnosed within 12 h of admission (*n* = 64); (4) recent exposure to iodinated contrast media (coronary angiography or contrast computed tomography) within 48 h prior to admission (*n* = 42); and (5) incomplete baseline or follow-up serum creatinine (SCr) records during the index hospitalization (*n* = 112). Consequently, the final analytical cohort comprised 1,071 patients.

### Outcome definition

The primary endpoint was new-onset AKI during the index hospitalization. AKI was defined according to modified KDIGO (48-h serum creatinine) criterion as an increase of at least 26.5 µmol/L within 48 h (Sarraf et al., 2009; Bellomo et al., 2012). The first available creatinine value at admission was used as the baseline measure. To address the lack of stable, pre-admission outpatient serum creatinine records in this retrospective cohort, the KDIGO 7-day relative-increase criterion (≥50% increase) was deliberately omitted. This conservative definition prevents the misclassification of preexisting chronic renal insufficiency or community-acquired, acute-on-chronic renal impairment as newly developed in-hospital events. To evaluate the robustness of this modified outcome definition, we performed a sensitivity analysis restricting the cohort to patients with a baseline eGFR ≥60 mL/min/1.73 m² to minimize the confounding effect of minor creatinine fluctuations in severe chronic kidney disease (CKD) stages. Urine-output criteria were also omitted due to incomplete urinary volume records in the electronic medical records database.

### Candidate predictors and leakage control

To keep the model applicable at first clinical contact, candidate predictors were restricted to variables available at hospital presentation. Admission eGFR was used as the marker of baseline renal function, whereas creatinine was not included in the final admission model to avoid collinearity with eGFR. To prevent chronological confounding and reverse causality, post-admission events, 48-h weight change, cumulative fluid balance, and inpatient loop-diuretic administration were excluded from model screening (Moons et al., 2015; Mullens et al., 2019). Hypotension on presentation was strictly defined as a documented systolic blood pressure (SBP) < 90 mmHg at first clinical contact, or the clinical requirement for immediate vasoactive or inotropic support within the first hour of hospital presentation. The furosemide-equivalent dose (expressed in mg/day) was strictly defined as the patient’s outpatient daily maintenance oral dose immediately prior to admission, converted to equivalents (furosemide 40 mg = torsemide 20 mg) to reflect baseline congestion severity and chronic treatment intensity.

### Statistical analysis and internal validation

Continuous variables are presented as medians with interquartile ranges (IQRs), and categorical variables as frequencies and percentages. Predictor selection was conducted using penalized logistic regression with cross-validation. To strictly prevent data leakage and optimism bias, feature screening was fully nested within a 5-fold stratified cross-validation framework. Candidate predictors were screened using the least absolute shrinkage and selection operator (LASSO) algorithm, with the regularization hyperparameter *λ* tuned via internal cross-validation according to the 1-standard error rule to enforce model parsimony. Selection frequencies of candidate variables across all five training folds were recorded to quantify feature stability. Predictors consistently selected across folds were subsequently refitted on the full dataset (*N* = 1,071) using multivariable logistic regression to derive the final regression coefficients, standard errors, odds ratios, and intercept. Model discrimination was evaluated using the area under the receiver operating characteristic curve (AUC-ROC) and precision-recall curve (PR-AUC). Probability accuracy and calibration were assessed via the Brier score, calibration slope, and intercept. Decision curve analysis (DCA) evaluated net clinical benefit. Statistical analyses were performed using R (version 4.2.2).

## Results

### Cohort characteristics and AKI incidence

Among the 1,071 included patients, 118 developed in-hospital AKI, corresponding to an incidence of 11.0%. Patients who developed AKI were generally older and showed a coherent admission profile of renal vulnerability, greater treatment intensity, and hemodynamic instability. Median baseline creatinine was higher in the AKI group [124.00 (96.00–158.00) vs. 95.00 (76.00–121.00) μmol/L], whereas median eGFR was lower [56.00 (41.00–71.00) vs. 69.00 (53.00–83.00) mL/min/1.73 m (Roger, 2021)]. Outpatient furosemide-equivalent dose was higher [125.50 (88.00–213.50) vs. 76.00 (50.00–113.00) mg/day], and hypotension on presentation was more frequent (24.6% vs. 4.4%). Baseline and on-admission characteristics are shown in **Table 1** and **Figure 2**.

**Table 1:** Baseline and on-admission characteristics by AKI status.

Variable	Overall (*N* = 1,071)	No AKI (*N* = 953)	AKI (*N* = 118)	*P*-value
Age, years	66.00 (56.00–74.00)	66.00 (56.00–74.00)	69.00 (60.00–76.75)	0.009
Height, cm	168.00 (165.00–172.00)	168.00 (165.00–171.00)	169.00 (165.25–173.00)	0.052
Weight, kg	72.00 (69.00–76.00)	72.00 (69.00–76.00)	73.00 (69.00–77.75)	0.040
BMI, kg/m^2^	25.51 (23.91–27.11)	25.51 (23.88–27.09)	25.54 (24.01–27.30)	0.545
Systolic blood pressure, mmHg	132.00 (119.00–147.00)	132.00 (119.00–147.00)	135.00 (120.00–146.75)	0.667
Diastolic blood pressure, mmHg	82.00 (74.00–91.00)	82.00 (74.00–91.00)	82.50 (72.00–92.00)	0.824
Heart rate, bpm	75.00 (67.00–84.00)	75.00 (67.00–83.00)	74.50 (66.00–85.00)	0.953
Pulse pressure, mmHg	50.00 (39.00–62.00)	50.00 (39.00–61.00)	50.00 (37.00–63.00)	0.706
Mean arterial pressure, mmHg	99.33 (90.00–109.00)	99.33 (90.00–109.00)	99.50 (91.00–111.92)	0.552
Rate-pressure product	9,882.00 (8,534.05–11,596.00)	9,882.00 (8,568.00–11,520.00)	9,911.00 (8,450.75–12,006.00)	0.765
Shock index	0.56 (0.49–0.66)	0.56 (0.49–0.66)	0.55 (0.48–0.67)	0.784
LVEDD, mm	65.00 (57.50–74.00)	65.00 (58.00–73.00)	64.50 (55.25–74.00)	0.527
LVEDD index	35.42 (31.24–40.28)	35.48 (31.30–40.39)	34.80 (29.96–39.73)	0.262
eGFR, mL/min/1.73 m^2^	68.00 (51.00–82.00)	69.00 (53.00–83.00)	56.00 (41.00–71.00)	<0.001
Creatinine, μmol/L	98.00 (78.00–126.00)	95.00 (76.00–121.00)	124.00 (96.00–158.00)	<0.001
Uric acid, μmol/L	299.14 (253.00–357.00)	298.08 (252.00–354.00)	312.50 (263.75–385.00)	0.044
Serum sodium, mmol/L	141.53 (133.49–150.65)	141.60 (133.42–151.17)	141.18 (134.46–146.74)	0.354
Serum potassium, mmol/L	4.44 (4.08–4.79)	4.45 (4.08–4.79)	4.37 (4.07–4.75)	0.253
White blood cell count	8.70 (6.90–10.70)	8.70 (6.90–10.70)	8.35 (6.72–10.60)	0.487
Neutrophil count	6.25 (4.54–8.27)	6.27 (4.58–8.31)	6.00 (4.25–8.00)	0.269
Lymphocyte count	1.50 (1.08–2.00)	1.50 (1.07–2.00)	1.50 (1.10–1.90)	0.969
Platelet count	204.00 (170.00–243.00)	203.00 (170.00–242.00)	207.00 (169.25–245.50)	0.657
RDW, %	12.70 (12.30–13.20)	12.70 (12.30–13.20)	12.80 (12.40–13.50)	0.034
High-sensitivity CRP	7.10 (2.20–25.90)	7.10 (2.20–25.90)	7.20 (2.60–25.46)	0.844
HbA1c, %	6.30 (5.70–7.14)	6.30 (5.70–7.15)	6.24 (5.58–7.05)	0.494
Fasting plasma glucose	5.64 (4.96–7.03)	5.65 (4.97–7.09)	5.59 (4.96–6.87)	0.618
Total cholesterol	4.29 (3.69–5.02)	4.26 (3.65–5.01)	4.47 (3.82–5.16)	0.093
Triglycerides	1.38 (1.01–1.98)	1.37 (1.00–1.99)	1.42 (1.06–1.90)	0.632
HDL cholesterol	0.96 (0.82–1.16)	0.96 (0.82–1.15)	0.96 (0.85–1.19)	0.666
LDL cholesterol	2.65 (2.12–3.23)	2.63 (2.11–3.21)	2.76 (2.22–3.31)	0.142
Lipoprotein(a)	218.00 (154.00–343.00)	218.00 (153.00–346.00)	208.00 (167.45–338.48)	0.868
Non-HDL cholesterol	3.30 (2.65–4.00)	3.27 (2.63–3.99)	3.44 (2.81–4.08)	0.133
Remnant cholesterol	0.58 (0.38–0.81)	0.58 (0.38–0.81)	0.60 (0.37–0.78)	0.969
TyG index	8.80 (8.42–9.20)	8.80 (8.41–9.21)	8.83 (8.47–9.14)	0.895
TyG-BMI	225.48 (207.24–243.69)	225.22 (207.11–243.93)	227.97 (209.74–242.57)	0.515
METS-IR	41.12 (37.74–45.01)	41.15 (37.63–45.12)	40.98 (38.30–43.99)	0.851
Log NT-proBNP	7.52 (6.76–8.27)	7.52 (6.74–8.25)	7.63 (6.96–8.44)	0.042
Albumin	38.20 (35.90–40.60)	38.20 (35.90–40.57)	37.60 (35.50–40.70)	0.422
PNI	45.60 (42.61–49.40)	45.70 (42.70–49.40)	45.00 (42.35–48.92)	0.319
GNRI	98.46 (95.08–102.13)	98.57 (95.16–102.07)	97.69 (94.32–102.30)	0.365
CKM burden	4.00 (3.00–5.00)	4.00 (3.00–5.00)	4.50 (3.00–6.00)	0.008
GDMT score	3.00 (2.00–3.00)	3.00 (2.00–3.00)	3.00 (3.00–3.00)	0.162
Furosemide-equivalent dose, mg/day	82.00 (52.00–119.00)	76.00 (50.00–113.00)	125.50 (88.00–213.50)	<0.001
Congestion score	2 (1–3)	2 (1–3)	3 (2–4)	<0.001
Male sex	788 (73.6%)	706 (74.1%)	82 (69.5%)	0.308
NYHA class III–IV	177 (16.5%)	142 (14.9%)	35 (29.7%)	<0.001
Smoking history	437 (40.8%)	390 (40.9%)	47 (39.8%)	0.820
Alcohol history	177 (16.5%)	157 (16.5%)	20 (16.9%)	0.896
Hypertension	276 (25.8%)	249 (26.1%)	27 (22.9%)	0.447
Diabetes mellitus	533 (49.8%)	461 (48.4%)	72 (61.0%)	0.010
Atrial fibrillation	471 (44.0%)	414 (43.4%)	57 (48.3%)	0.315
Moderate/high GNRI risk	99 (9.2%)	85 (8.9%)	14 (11.9%)	0.297
MRA use	775 (72.4%)	686 (72.0%)	89 (75.4%)	0.431
CCB use	122 (11.4%)	103 (10.8%)	19 (16.1%)	0.088
Hypotension on presentation	71 (6.6%)	42 (4.4%)	29 (24.6%)	<0.001

*P*-values are descriptive group comparisons and were not used as automatic model-entry rules.

BMI, body mass index; BP, blood pressure; MRA, mineralocorticoid receptor antagonist; CCB, calcium channel blocker; IV, intravenous.

**Figure 2:**
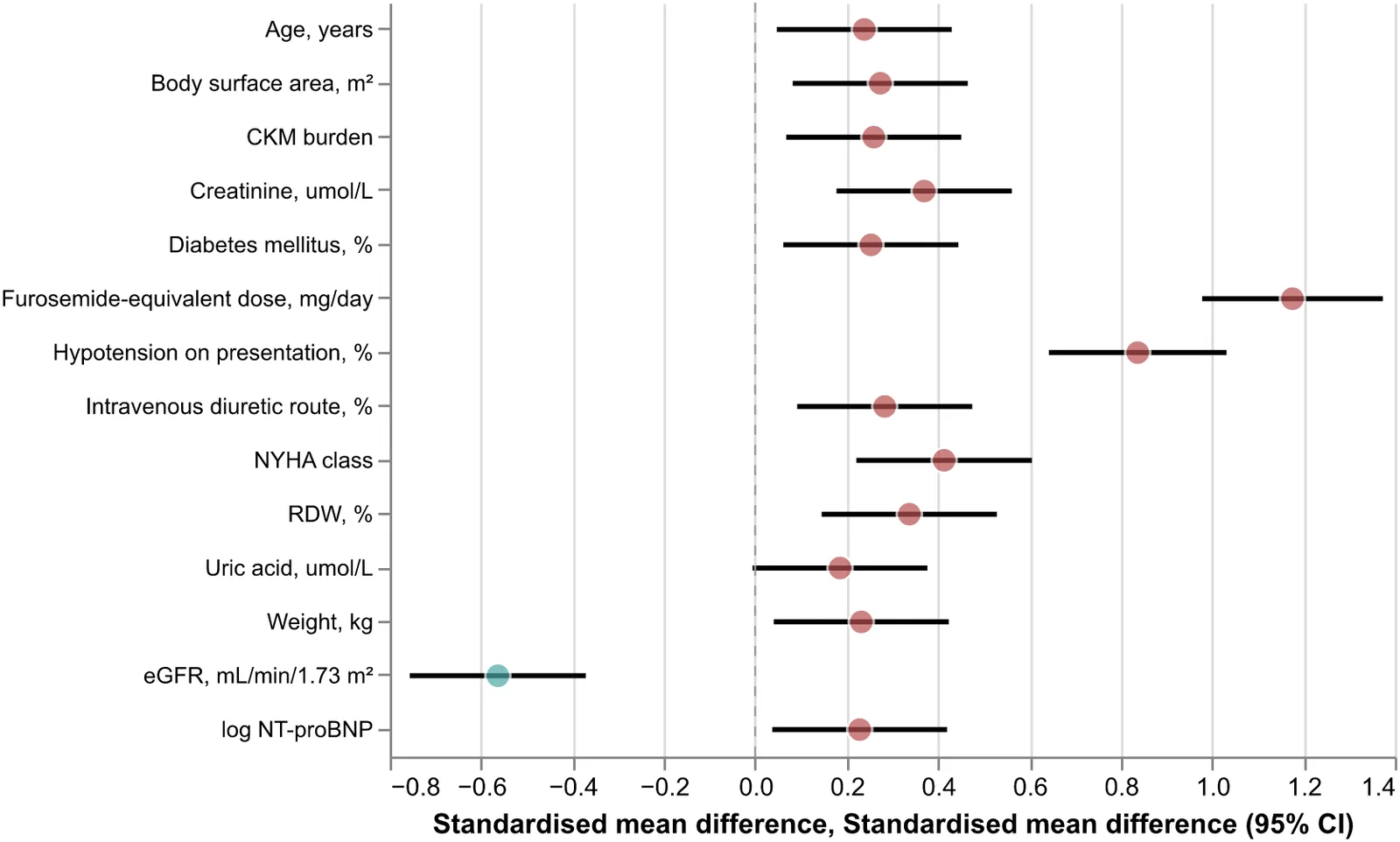
Clinical differences between patients with and without in-hospital AKI.

### Algorithmic screening produced a five-variable on-admission model

Following the selection of eligible on-admission parameters based on clinical criteria, 13 candidate predictors available at presentation were evaluated through the nested LASSO pipeline. Across all five cross-validation training folds, exactly five predictors achieved a 100% fold selection frequency: age, CKM burden, creatinine, diabetes mellitus, furosemide-equivalent dose, hypotension on presentation, intravenous diuretic route, NYHA class, RDW, uric acid, weight, eGFR, and log NT-proBNP. Variables known to contain endpoint information, information unavailable at admission, or redundant renal function information had already been removed and were not offered to the algorithm. The final primary admission model retained five predictors: age, furosemide-equivalent dose, hypotension on presentation, RDW, and eGFR (**Table 2****;** **Figure 3**).

**Table 2:** Predictor eligibility and algorithmic screening rationale for the primary clinical model.

Admission-eligible variable	Entered algorithmic screen	In the primary model	Rationale
Age	Yes	Yes	Retained: baseline vulnerability marker
CKM burden	Yes	No	Screened but not retained: comorbidity/metabolic signal overlapped with retained variables
Creatinine	Yes	No	Screened but not retained: renal function signal adequately captured by eGFR under redundancy constraint
Diabetes mellitus	Yes	No	Screened but not retained: comorbidity signal overlapped with retained variables
Furosemide-equivalent dose	Yes	Yes	Retained: admission treatment-intensity marker selected over IV_Diuretic by algorithmic screening
Hypotension on presentation	Yes	Yes	Retained: presentation-window hemodynamic stress marker
Intravenous diuretic route	Yes	No	Screened but not retained: diuretic treatment signal adequately captured by Furosemide_Eq_Dose under redundancy constraint
NYHA class	Yes	No	Screened but not retained: hemodynamic/HF severity signal captured by other retained variables
RDW	Yes	Yes	Retained: inflammation/anemia marker
Uric acid	Yes	No	Screened but not retained: renal vulnerability adequately captured by eGFR
Weight	Yes	No	Screened but not retained: body-size signal did not improve AUC beyond the selected set
eGFR	Yes	Yes	Retained: objective renal function marker; selected over Creatinine and Uric_Acid by algorithmic screening under redundancy constraint
log NT-proBNP	Yes	No	Screened but not retained: HF severity signal overlapped with retained variables

**Figure 3:**
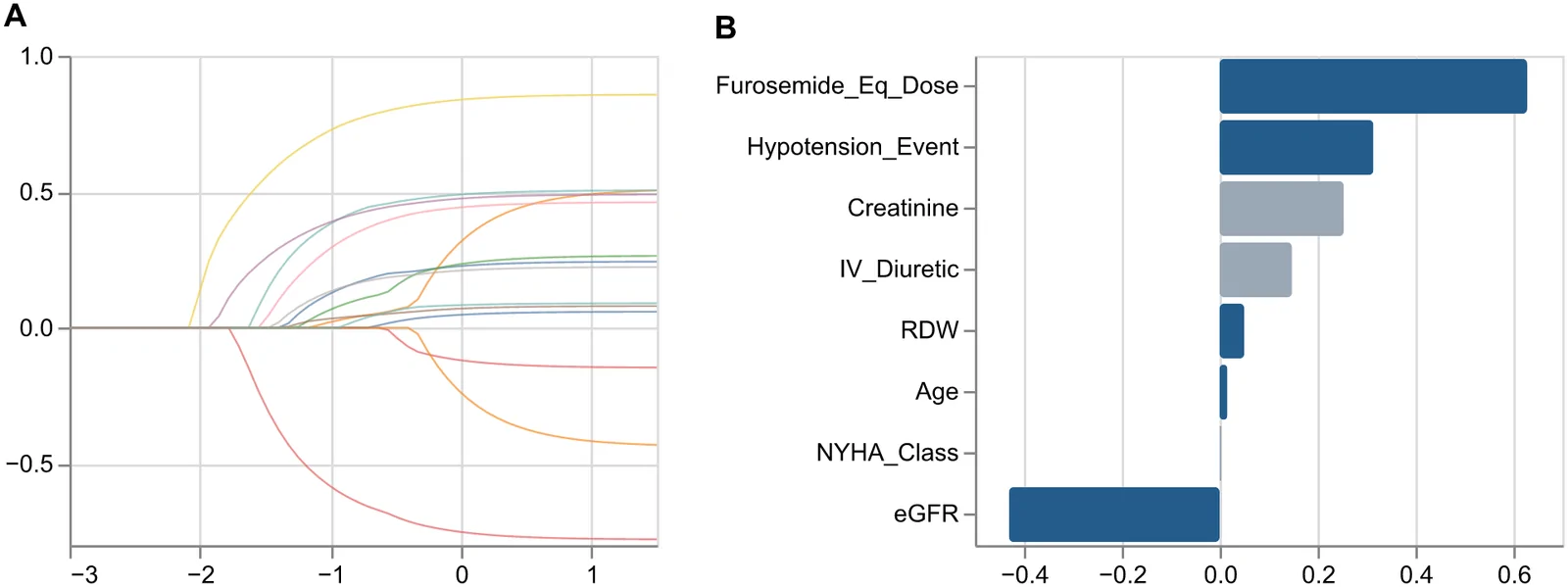
**(A)** LASSO regression coefficient profiles of candidate predictors across varying regularization parameters; **(B)** Selected predictor variables and their corresponding model coefficients.

This selection strategy was chosen to avoid post-admission predictors, overfitting, and redundant physiology. With 118 AKI events, the five-variable model achieved approximately 23.6 events per predictor, supporting a conservative admission-only modeling stance for a single-center retrospective dataset.

### Multivariable model specification and prediction equation

The multivariable logistic regression model refitted on the complete cohort (*N* = 1,071) yielded the following final prediction equation for individual AKI probability: logit(*P*) = 1/(1 + e^(6.0430 − 0.0229 × Age − 0.0127 × Furosemide_Eq_Dose − 1.7431 × Hypotension_Event − 0.3475 × RDW + 0.0386 × eGFR)). Exact regression coefficients, standard errors, odds ratios, and 95% confidence intervals are reported in **Table 3**.

**Table 3:** Univariate and multivariable logistic regression analyses of factors associated with AKI.

Variable	Univariate analysis		Multivariable analysis				
	OR (95% CI)	*P*-value	*β*	SE	Wald *z*	OR (95% CI)	*P*-value
Age (per year)	1.020 (1.004–1.037)	0.015	0.0229	0.0094	2.435	1.023 (1.004–1.042)	0.015
Furosemide-equivalent dose (per mg)	1.013 (1.010–1.016)	<0.001	0.0127	0.0015	8.569	1.013 (1.010–1.016)	<0.001
Hypotension on presentation (yes vs. no)	7.068 (4.198–11.899)	<0.001	1.7431	0.3175	5.489	5.715 (3.067–10.648)	<0.001
RDW (per %)	1.477 (1.180–1.849)	<0.001	0.3475	0.1298	2.676	1.415 (1.097–1.826)	0.007
eGFR (per mL/min/1.73 m^2^)	0.968 (0.958–0.979)	<0.001	−0.0386	0.0066	−5.869	0.962 (0.950–0.975)	<0.001
Intercept (constant)			−6.0430	1.8797	−3.215		0.001

OR, odds ratio; CI, confidence interval; RDW, red cell distribution width; eGFR, estimated glomerular filtration rate.

### Temporal onset breakdown and subgroup sensitivity analysis

Regarding temporal onset among the 118 AKI cases, 84 (71.2%) occurred within 0 to 24 h of admission, 26 (22.0%) occurred within 24 to 48 h, and 8 (6.8%) occurred beyond 48 h post-admission, demonstrating that the 48-h diagnostic window successfully captured 93.2% of early in-hospital AKI events directly tied to baseline presentation.

In the prespecified sensitivity analysis restricted to patients with preserved baseline renal filtration capacity, 49 patients experienced in-hospital AKI (7.1% incidence). The five-variable on-admission model preserved high discriminatory performance and probability calibration within this subcohort, achieving an AUC-ROC of 0.79 (95% CI 0.73–0.84), a precision-recall AUC of 0.38, a Brier score of 0.06, a calibration slope of 0.96, and a calibration intercept of −0.01.

### Internal validation, calibration, and clinical utility

In five-fold stratified out-of-fold internal validation, the primary admission model achieved an AUC-ROC of 0.81 (95% CI 0.77–0.85) and a PR-AUC of 0.47 (bootstrap 95% CI 0.34–0.53). Probability accuracy was acceptable, with a Brier score of 0.08 and log loss of 0.28. At the selected operating point, sensitivity was 0.43, specificity 0.93, positive predictive value 0.43, and negative predictive value 0.93 (**Table 4** **and** **Figure 4**).

**Table 4:** Internal validation performance of the primary admission model.

Model	Vars	AUC	PR-AUC	Brier	Cal slope	Sens	Spec	PPV	NPV
Model	5	0.81	0.47	0.08	0.98	0.43	0.93	0.43	0.93

Metrics are from 5-fold stratified out-of-fold internal validation.

**Figure 4:**
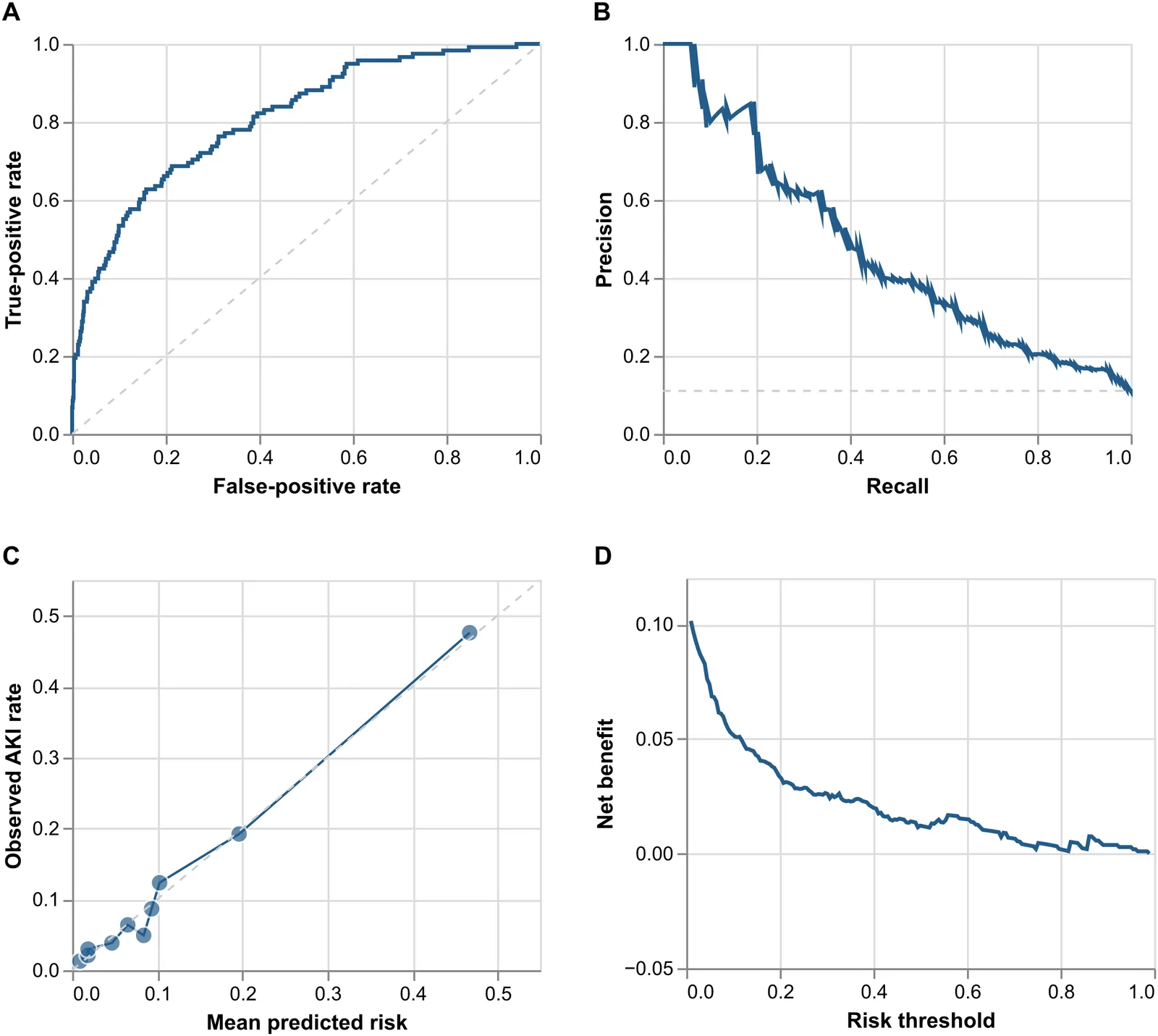
**(A)** Receiver operating characteristic (ROC) curve showing model discrimination; **(B)** Precision-recall (PR) curve; **(C)** Calibration plot displaying mean predicted risk versus observed AKI rate; **(D)** Decision curve analysis (DCA) evaluating clinical net benefit across different risk thresholds.

The decision curve analysis suggested potential net benefit in internal validation; however, this requires external validation before clinical use. Risk stratification was clinically monotonic at the extremes: observed AKI was 1.9% in the lowest predicted risk quintile and 35.5% in the highest predicted risk quintile. The middle quintiles showed some local non-monotonicity, reinforcing the need for external validation before individual-level clinical deployment.

### Benchmark and alternative model comparisons

Alternative clinical specifications were evaluated to test whether the chosen primary baseline variables were overly simplified. Head-to-head model comparison demonstrated that the primary eGFR-based model achieved superior discrimination (AUC = 0.81, 95% CI 0.77–0.85) compared with the serum creatinine-based model (AUC = 0.79, 95% CI 0.75–0.83). The eGFR-based specification also demonstrated improved reclassification tendency, yielding a continuous net reclassification improvement (NRI) of +0.041 (*P* = 0.12).

## Discussion

This study developed and validated a parsimonious five-variable on-admission risk scoring system for predicting new-onset in-hospital AKI in heart failure patients with reduced ejection fraction (HFrEF). In contemporary clinical practice, characterized by robust neurohormonal therapies such as sodium-glucose cotransporter 2 inhibitors (McMurray et al., 2019; Anker et al., 2021), early cardiorenal risk stratification remains critical. Instead of relying on complex, overparameterized algorithms incorporating post-admission kinetics or dynamic biomarkers (Damman et al., 2007; Moons et al., 2015; Moons et al., 2019), our system relies strictly on easily accessible on-presentation parameters: eGFR, loop diuretic dose, hypotension on admission, red cell distribution width (RDW), and age. Compared with established risk tools evaluated in the same cohort—such as conventional clinical risk scores like ADHERE/GWTG-HF (Aykut et al., 2026) (AUC = 0.55, 95% CI 0.53–0.57), our admission-based model demonstrated significantly superior discriminatory performance (AUC = 0.81, 95% CI 0.77–0.85).

These variables represent the fundamental baseline requirements for evaluating patients at presentation. The five predictors directly map onto the key pathophysiological axes of cardiorenal interaction in HFrEF (Ronco et al., 2010). Advanced age represents a major driver of physiological senescence, characterized by vascular calcification, loss of arterial compliance, and cellular-level nephropathy (Coca et al., 2009; Denic et al., 2016). When acute hemodynamic stressors occur, older kidneys are highly vulnerable to ischemic insult due to pre-existing glomerulosclerosis and interstitial fibrosis (Hoste et al., 2018).

The incorporation of eGFR and RDW provides integrated surrogates for systemic homeostasis and renal reserve (Mullens et al., 2009). In patients with HFrEF, baseline renal filtration capacity is a direct measure of functional nephron mass (Bellomo et al., 2012; Chawla et al., 2017). Patients with a compromised baseline eGFR operate on the edge of their physiological compensation, possessing minimal reserve to buffer systemic hypotension or aggressive pharmacotherapy (Damman et al., 2009; Chawla et al., 2017). Meanwhile, RDW serves as an elegant, integrated surrogate marker reflecting systemic chronic inflammation, iron metabolism disruption, oxidative stress, and hyperactivation of neurohormonal pathways in heart failure (Ambrosy et al., 2013; ter Maaten et al., 2015). Systemic chronic inflammation and oxidative stress downregulate renal microvascular endothelial function, severely impairing renal blood flow autoregulation and predisposing the renal parenchyma to acute ischemic tubular injury even during minor hemodynamic fluctuations (Smith et al., 2006). Hypotension on admission represents acute hemodynamic perfusion failure, directly starving the oxygen-sensitive thick ascending limb of Henle in the outer renal medulla (ter Maaten et al., 2015). Simultaneously, the initial furosemide-equivalent loop diuretic dose reflects presenting congestion severity and treatment intensity (Felker et al., 2011; Mullens et al., 2019), which introduces acute intravascular volume contraction and reflexive sympathetic activation (ter Maaten et al., 2015).

This five-variable model offers a clear, structured application path in bedside clinical triage. Clinicians can immediately compute the AKI probability using on-admission parameters. For patients identified as high risk, a proactive renal protective pathway is initiated: this includes ordering daily serum creatinine and electrolyte monitoring, strictly avoiding non-steroidal anti-inflammatory drugs (NSAIDs) or other nephrotoxic agents, implementing close hourly fluid balance tracking, and adopting a gradual, stepwise, or urine-sodium-guided diuretic strategy to avoid precipitating intravascular volume depletion. Conversely, patients identified as low risk represent a cohort that might possess greater physiological reserve and be less prone to precipitate acute renal decline. This stratification may support clinicians in pursuing guideline-recommended, timely decongestion with close hemodynamic monitoring. However, our model is not intended to automate or dictate therapeutic decisions. All diagnostic predictions must be integrated with real-time clinical judgment, as worsening renal function during decongestion does not always equate to structural tubular damage but still warrants close attention (Mullens et al., 2022). This dual-track approach ensures that risk prediction serves as an adjunctive decision-support tool for tailored, patient-specific heart failure management, rather than a rigid therapeutic protocol.

Several limitations must be addressed to contextualize our findings. This was a single-center retrospective study lacking external validation in independent geographic or community hospital cohorts. Prospective multicenter external validation is essential before clinical deployment. Crucially, true outpatient baseline creatinine values during periods of stable clinical state were unavailable for the majority of our historical cohort (Bellomo et al., 2012). Utilizing the first available admission creatinine as the baseline renal metric represents a noteworthy limitation, as this value may already be pathologically elevated due to acute hemodynamic congestion, potentially leading to underdiagnosis of community-acquired AKI or over-indexing of GFR (Long et al., 2019). Additionally, we lacked precise, hourly timestamps for AKI onset, preventing dynamic Cox proportional hazards analysis (Stoltzfus, 2011). There were 112 patients who were excluded due to incomplete serum creatinine records, which may introduce selection bias inherent to retrospective data collection. Finally, we could not incorporate novel urinary renal injury biomarkers (NGAL or KIM-1) as they are not routinely measured (Chawla et al., 2017; Hoste et al., 2018). Prospective multicenter external validation and implementation impact assessments are required before this tool is integrated into routine clinical decision-making.

## Conclusion

A five-variable model based on eGFR, outpatient loop-diuretic dose, hypotension at admission, RDW, and age provided internally validated risk stratification for new-onset in-hospital AKI among patients hospitalized with HFrEF. The model was designed to be clinically interpretable and to avoid variables that are unavailable at admission or already contain endpoint information. Prospective multicenter validation, protocolized predictor measurement, and implementation-impact assessment are required before the model is used to guide routine clinical decisions.

## Data Availability

The datasets generated and/or analyzed during the current study are not publicly available due to patient privacy and institutional ethical restrictions, but are available from the corresponding author on reasonable request. Requests to access the datasets should be directed to wangyidf1019@163.com.
